# The impact of social media addiction on the negative emotions of adolescent athletes: the mediating role of physical appearance comparisons and sleep

**DOI:** 10.3389/fpubh.2024.1452769

**Published:** 2025-01-24

**Authors:** Weilong Lin, Zhenyu Cen, Ying Chen

**Affiliations:** ^1^Sports Training Academy, Guangzhou Sport University, Guangzhou, China; ^2^School of Physical Education and Sports Science, South China Normal University, Guangzhou, China; ^3^School of Foreign Languages and Culture, Guangdong University of Finance, Guangzhou, China

**Keywords:** adolescent athletes, social media addiction, negative emotions, physical appearance comparison, sleep quality

## Abstract

Extensive use of social media is commonly associated with the development of social media dependency and amplification of adverse emotions among adolescent athletes. Descriptive statistics, correlation analysis, and path analysis were employed to investigate the impact of social media addiction on the negative emotions experienced by adolescent athletes. This study included a sample of 362 adolescent athletes (aged 14–21) participating in various sports events at training facilities in Guangdong and Hunan. Data collection was performed using the Social Media Addiction Scale, Body Appearance Comparison Scale, Pittsburgh Sleep Quality Index (PSQI), and the Depression, Anxiety, and Stress Scale (DASS-21). The findings suggest that social media addiction contributes directly to heightened negative emotions in adolescent athletes. In addition, physical appearance comparisons and sleep quality were identified as mediating factors that intensify the influence of social media addiction in terms of negative emotions. Consequently, it is recommended to underline the importance of mental health support for adolescent athletes, implement effective strategies for managing social media use, promote healthy body image perceptions, enhance sleep quality, and address negative emotions among adolescent athletes.

## 1 Introduction

The use of social media is experiencing widespread growth globally (1), including in China. Recent data from the Digital 2021: Global Overview Report indicates a 1.5-fold increase in social media use over the past 5 years (2). Prominent social networks such as Facebook, YouTube, and WhatsApp are widely used worldwide, and platforms such as WeChat and Weibo are popular in China. Social media addiction has evolved from a limited concern to a condition recognized as a global epidemic, with individuals across the globe displaying excessive engagement and investing substantial time on social media platforms (3, 4).

This excessive usage has been found to have adverse effects on the lives of millions of individuals worldwide (4). In this digital information era, social media has become an integral aspect of the daily routines of young people, with a noticeable uptrend in its popularity and the time spent on these platforms (2). The rise in psychological issues due to social media addiction significantly hinders the development of well-rounded individuals excelling in morality, intelligence, physical health, and artistry (5). Adolescents’ mental health challenges are multifaceted, with social media addiction identified as a key contributing factor (6). Young athletes routinely use social media to share training progress, exchange competition insights, and access fitness-related information. However, excessive use of social media and addiction problems have prevented them from reaching competitive peaks, taking up their time and negatively impacting their mental well-being, particularly in managing adverse emotions. The inundation of information and frequent interactions on social media platforms can lead to heightened comparisons of physical appearance among young athletes, potentially diminishing self-worth and fostering negative emotions (7). Excessive social media consumption can disrupt sleep patterns and erode the quality of sleep of adolescent athletes, which is crucial for emotional regulation. Social media addiction can intensify negative emotions in adolescent athletes through the double mediating effect of physical appearance comparisons and sleep quality. Therefore, investigating the influence of social media addiction on negative emotions in adolescent athletes and exploring the mechanisms of action is essential for comprehending the pathways of social media addiction on their mental health. The study is also constructive in elucidating the mediating roles of physical appearance comparisons and sleep in the relationship between social media addiction and negative emotions, providing precise instructions and insightful perspectives for managing and enhancing the mental well-being of adolescent athletes. This study is expected to be a valuable resource for promoting healthy development and mental resilience, thereby contributing positively to the advancement of youth sports and informing the development of effective intervention strategies based on the available scientific evidence.

## 2 Related concepts and research hypotheses

### 2.1 Investigating the impact of social media addiction on the experience of adverse emotions among adolescent athletes

Social media encompasses the use of electronic devices and networks as platforms for the dissemination, generation, and interchange of ideas, data, videos, and images within virtual networks and communities, exemplified by platforms such as Weibo and WeChat (8). In the contemporary information era, social media has become an integral component of societal interactions. Previous research indicates that 93% of teenagers engage with social media daily, with 56% expressing an inability to envision life without a smartphone (9). The pervasive influence of social media adversely affects the lives of numerous individuals globally, who exhibit symptoms such as salience, conflict, tolerance, withdrawal, relapse, and altered moods (10). These manifestations result in diminished sleep quality, excessive spiritual occupancy, persistent thoughts of excessive and unregulated use, and the inability to control the duration of social media use (11). Notably, realistic socialization capabilities are diminished, and the aptitude for discerning emotions in others is compromised (12), potentially culminating in mental health issues such as anxiety disorders (4), schizophrenia (13), eating disorders (14, 15), personality disorders (16), and substance use disorders (17). Social media addiction can induce adverse emotional states in individuals. Emotion is characterized as an individual’s subjective perceptual state, representing a transient pleasant or unpleasant condition (18), an emotional reaction within a sequence of encounters, an interpretive awareness of behavior, and a spiritual process intertwined with the present temporal and spatial context (19). Negative emotions, typified by anger, sadness, and fear, can persist for a prolonged duration and prove challenging to manage (20), leading to conditions such as eating disorders and exerting an impact on individuals’ daily routines, health, and subjective well-being (15). The prevalence of negative emotions can impact subjective happiness and the overall quality of life considerably, with social media addiction identified as a primary instigator of such adverse emotional states (21). The ramifications of social media addiction on adolescent populations predominantly take in the physical implications (e.g., sleep disturbances and physical harm), psychological effects (cognitive impacts, emotional ramifications, and subjective well-being), and behavioral outcomes (academic performance, professional efficacy, social interaction, and sporadic use of social media) (22), fostering anxiety, stress, and apprehension among adolescents (21). Notably, studies have underscored the heightened vulnerability of athletes to certain facets of mental health challenges attributed to diverse factors such as their lifestyle, athletic training (including financial performance), and physical appearance (particularly weight management and related concerns) (23). Thus, building on these insights, this study posits Hypothesis 1: The intensity of social media addiction correlates positively with the magnitude of negative emotional repercussions experienced by adolescent athletes.

### 2.2 Moderating effect of physical appearance comparison

The concept of physical appearance comparison includes an individual’s continuous assessment of their physical characteristics in relation to a specific standard (24). Social media has emerged as a prominent platform for adolescents to compare physical appearance, particularly in the context of the differential susceptibility to media effects model (25). Excessive use of social media triggers cognitive, emotional, and excitatory responses that influence mental health regulation. An individual’s media consumption and subsequent reactions are deeply intertwined with their surroundings, which are notably shaped by temperamental characteristics, developmental stages, and social influences. Research by Gerwin et al. indicates that self-blame for physical appearance due to digital social media use (26), along with persistent comparison with and emulation of unrealistic role models (27), can lead to continuous upward social comparisons that motivate individuals. However, this negative cognitive self-assessment can result in dissatisfaction, depression, and self-deprecation (28, 29). The widespread adoption of social media has intensified the frequency of physical appearance comparison (6, 30), providing adolescents with a convenient avenue for such evaluation. Consequently, social media use can facilitate physical appearance comparisons. Social comparison theory underscores that comparing oneself to others based on physical appearance carries the inherent risk of body dissatisfaction (7). According to this theory, pressure from influential social entities such as peers, family, and media can lead to body dissatisfaction and adherence to culturally endorsed appearance standards, such as an ideal body shape for women and a muscular physique for men. These standards are believed to be attained through the internalization of appearance ideals and appearance-based social comparisons (31). Previous research has demonstrated a substantial correlation between the inclination to compare physical appearance and various factors including self-esteem, anxiety levels, sexual satisfaction, a drive for muscular development, compulsive exercise, and body dysmorphia. Compulsive exercise, and body dysmorphic disorder (32–34). This correlation often results in diminished self-esteem, heightened anxiety, depression, and other adverse emotions (6). Building on these findings, this study posits the following research hypothesis H2: The mediating role of body appearance comparison in the impact of social media addiction on negative emotions among adolescent athletes.

### 2.3 Mediating effect of sleep quality

Increased social media use is thought to be associated with decreased sleep quality, and poor sleep quality contributes to daytime sleepiness in adolescents (35), negatively affecting their performance, academic achievement, activity levels, and energy (36). Social media addiction and nighttime social media use have a multifaceted impact on health (37). A study of British teenagers found that excessive nighttime use of social media led to 20% of teenagers experiencing fatigue, fear of missing out, and reduced sleep quality (38). The importance of sleep among athletes is considered even more important than that of the general population (39). Problems with sleep quality and quantity are common among athletes, often due to time conflicts or the physiological effects of training (39, 40). Charest and Grandner emphasize that sleep deprivation leads to a variety of physical and psychological consequences, such as heightened stress, anxiety, and a diminished capacity to cope with such stress and recuperate effectively. Therefore, sleep problems may represent another area of vulnerability for athletes’ mental health. According to these studies, sleep patterns are strongly correlated with those of non-athletes, adolescents, and adult athletes. Training and competition schedules, heightened pre-game arousal levels, and delayed sleep onset caused by the use of electronic devices before bedtime are frequently the primary factors that impact athletes’ sleep. These factors are generally associated with reduced sleep quality and quantity, which are attributed to emotional responses to digital and social media stimuli. Sleep has a more substantial impact on athletes than non-athletes. Previous studies have suggested that athletes are prone to experiencing insomnia symptoms before major competitions, during high-intensity training, and after long-distance travel. Fatigue and anxiety caused by poor sleep impact training and competition directly (41). Sleep duration and quality are closely linked to negative emotions, with poorer sleep quality showing a stronger correlation with negative emotions (42). Compared with athletes who have good sleep quality, athletes with poor sleep quality exhibit lower levels of consciousness and working efficiency, and are thereby more prone to experiencing anxiety (43). Based on this, this study proposes research hypothesis H3: Sleep quality plays a mediating role in the influence of social media addiction on the negative emotions of young athletes.

### 2.4 Chain mediation of body appearance comparison and sleep quality

The aforementioned analysis suggests that negative mood is influenced by social media addiction, body appearance comparison, and sleep quality. In addition, social media addiction impacts both physical appearance comparison and sleep quality. Studies indicate that a substantial relationship exists between physical appearance comparison and sleep quality, with the former directly affecting the latter (44), directly resulting in appearance-related anxiety, eating disorders, and sleep disturbances (24). Comparison of body appearance is linked to the development of body image and sleep disorders (45). Furthermore, body appearance comparison has been shown to affect sleep quality. Isabel et al. discovered that physical appearance comparisons directly impact sleep quality, and positive comparisons of body image can improve sleep quality (46). The prevalence of social media has shifted the focus of physical appearance comparison from real life to online platforms, particularly with the widespread use of beautification software, which intensifies the anxiety associated with comparing physical appearance (30). Building on the aforementioned research findings, this study posits hypothesis H4: Physical appearance comparison and sleep quality act as chain mediators in the negative emotional consequences of social media addiction on adolescent athletes.

**Figure 1:**
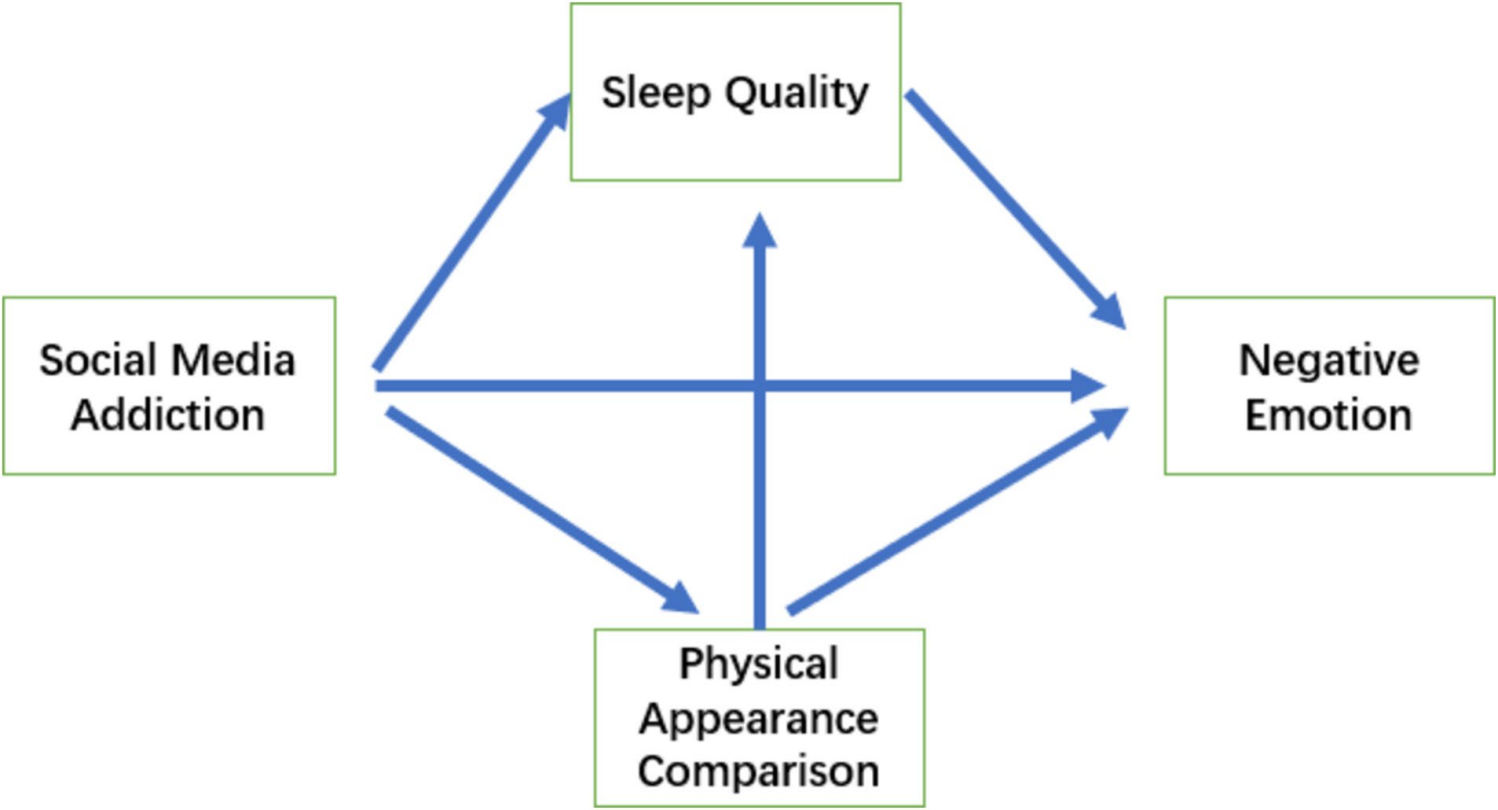
Theoretical model diagram.

See Figure 1 for the detailed research model diagram.

## 3 Research design

### 3.1 Research sample selection

This study used the online survey platform Wenjuanxing for data collection. A three-part questionnaire was designed to capture the demographic characteristics, social media addiction, and negative emotions of the participants. The estimated completion time of the questionnaire is 20 min. Because of China’s three-level training system for competitive sports, provincial training bases attract young athletes nationwide. These athletes have a solid foundation in sports and training and are pursuing professional development, thus forming the cornerstone of high-level competitive sports in China. The focus of this paper is on young athletes at the training bases in Guangdong and Hunan. Before participants joined the study, detailed research information was provided to the athletes and coaches, and their informed consent was obtained. Some studies suggest that adolescents aged 14 years or older are considered capable of consenting to participate in the study without parental permission (47). For participants under 14 years of age, parental consent was required and obtained using an online form. The study design and consent procedure were approved by the Institutional Ethics Committee. Because all data were collected anonymously, no direct link could be made between participants and mental health indicators. At the end of the data collection, participants were given access to a personalized feedback report that highlighted high-risk values and included contact information for the mental health crisis assistance agencies available to all participants. The data collection was scheduled to take place between September and October 2023. Before data collection, the Free Statistics Calculator V.4.0 was used in this paper to establish the 95% confidence interval (i.e., squared multiple correlations) and calculate that the sample size for this study should not be less than 238. Participants aged 14 to 19 years were selected for this study. Questionnaires that fell outside this age range or provided non-credible information (e.g., non-existent sports or unrealistic online activities or hours of use) were excluded.

### 3.2 Sample research

A total of 362 athletes were initially chosen for inclusion in the survey, with 320 individuals meeting the criteria for age, event, and other pertinent factors, thus forming the final sample for analysis. The demographic characteristics of the sample are detailed in Table 1, which includes participants from 21 distinct sports. Notably, most participants, constituting 72% of the sample, engage in one of the top six sports: swimming (17%), soccer (16%), track and field (15%), hockey (9%), ice hockey (9%), and basketball (6%). This study prioritizes athletes’ competitive performance levels as the crucial criterion for assessing their sporting achievements. The participants were categorized into three groups based on the performance standards of Chinese athletes: master level (comprising international masters of sports and master of sports), professional level (including first-level and second-level athletes), and amateur level (representing young athletes who have yet to be formally designated as such).

**Table 1:** Descriptive statistics of survey data.

Variable	Mean value (*n* = 320)	Male (*n* = 198)	Female (*n* = 122)	Master (*n* = 45)	Professional (*n* = 90)	Amateur (*n* = 185)
Age (year)	16.15 ± 3.86	16.02 ± 3.69	16.369 ± 4.133	23.09 ± 4.64	15.92 ± 1.62	14.57 ± 2.28
Training per week (hour)	24.57 ± 10.44	23.64 ± 9.47	26.10 ± 11.73	30.78 ± 7.96	22.63 ± 8.86	24.01 ± 11.15
Time spent on social media per week (hour)	11.00 ± 9.00	10.45 ± 8.59	11.89 ± 9.60	18.19 ± 12.46	10.93 ± 8.07	9.28 ± 7.50
Social media addiction	39.05 ± 4.56	38.61 ± 4.86	39.77 ± 3.93	39.29 ± 5.04	39.82 ± 4.09	38.62 ± 4.62
Stresses	10.08 ± 3.10	9.77 ± 2.91	10.58 ± 3.31	10.20 ± 3.45	9.68 ± 2.78	10.24 ± 3.15
Anxiety	10.75 ± 3.70	10.22 ± 3.47	11.61 ± 3.90	10.42 ± 4.02	10.72 ± 3.49	10.84 ± 3.73
Depression	9.20 ± 3.29	8.81 ± 3.18	9.84 ± 3.37	9.11 ± 3.11	8.67 ± 2.64	9.49 ± 3.58
Das-21	30.03 (9.22)	28.80 ± 8.70	32.02 ± 9.71	29.73 ± 9.88	29.07 ± 8.17	30.57 ± 9.54
Body comparison scale	11.72 ± 4.83	11.51 ± 5.00	12.06 ± 4.53	11.73 ± 4.70	11.49 ± 4.87	11.83 ± 4.86
Pittsburgh sleep index	3.84 ± 1.62	3.42 ± 1.42	4.53 ± 1.70	4.52 ± 1.59	3.81 ± 1.66	3.69 ± 1.58

The average (standard deviation) of the total sample is calculated by gender and performance level.

### 3.3 Data scales and measurements

This research focuses on investigating the influence of digital media on the psychological well-being of adolescent athletes. Scholars such as Nixdorf (48) and Mancine (49) assert that mental health concerns can manifest as negative emotions and eating disorders. Negative emotions are recognized as a crucial aspect of mental health within the framework of hedonic emotions. Consequently, this study is dedicated to investigating related research from the perspective of negative emotions and eating disorders as symptoms of deteriorating mental health.

#### 3.3.1 Depression, anxiety, and stress scale

In this investigation, negative emotions were selected as a parameter to explore the vulnerability of athletes to stress-related mental health challenges. The study employed the Depression Anxiety and Stress Scale 21 (DASS-21) to assess negative mental health emotions among adolescent athletes (50, 51). The DASS-21 is a comprehensive 42-item instrument comprising three distinct subscales designed to evaluate depression, anxiety, and stress, with each subscale consisting of seven items (50). Prior research has indicated that the DASS-21 and its subscales exhibit strong reliability in adolescent populations, a trend that has been observed to be consistent across various age brackets and genders (52). The current study found that DASS-21 displayed notably high internal consistency, with an overall $\mathrm{Cronbac}h^{\prime }s\;\alpha =0.93$ . The individual subscales demonstrated $\mathrm{Cronbac}h^{\prime }s\;\alpha$ coefficients ranging from 0.81 to 0.89. Given the blended findings in existing literature regarding the factor validity of using DASS-21 subscales versus the total scale in adolescent populations (52), this study decided to use the total scale of DASS-21 as a metric for assessing mental health impairment in adolescents.

#### 3.3.2 Sleep quality index

The Pittsburgh Sleep Quality Index (PSQI) was employed as a measurement tool in this study. This index is globally acknowledged for assessing sleep quality (53) and comprises 19 test indicators and seven data dimensions. A higher PSQI score indicates a more severe sleep disorder (54). A meta-analysis revealed that PSQI exhibited internal consistency with a $\mathrm{Cronbac}h^{\prime }s\;\alpha$ coefficient of 0.7, demonstrating strong structural reliability. In the present study, PSQI displayed internal consistency with a $\mathrm{Cronbac}h^{\prime }s\;\alpha$ coefficient of 0.73, indicating a high level of reliability.

#### 3.3.3 Social comparison scale

Social comparison uses the Physical Appearance Comparison Scale (PACS), which is a broadly employed tool for assessing individuals’ tendencies to habitually engage in comparisons related to physical appearance. Comprising five items, the scale prompts participants to evaluate the extent to which they compare their physical attributes with those of others using a five-point Likert scale. The reliability of PACS is $\mathrm{Cronbac}h^{\prime }s\;\alpha =0.7$ (55). Among adolescents, the reliability of PACS is $\mathrm{Cronbac}h^{\prime }s\;\alpha <0.7$ , but with particular attention to challenges arising from the interpretation of item 4. This item was misconstrued by young respondents because of its focus on broad attitudes rather than specific behaviors, leading to increased levels of abstraction and confusion and a low degree of matching stemming from frequency-based response options (e.g., “never,” “always”). This complexity may pose difficulties in comprehension among young teenagers. Excluding the fourth item can enhance the reliability of PACS, which can be $\mathrm{Cronbac}h^{\prime }s\;\alpha =0.79$ (55). To obviate the risk of obtaining unrealistic outcomes, this study opts for four variations of PACS to assess its reliability and the reliability of PACS is $\mathrm{Cronbac}h^{\prime }s\;\alpha =0.84$ .

#### 3.3.4 Digital media addiction

This study employs the Gaming Disorder Scale for Adolescents (GADIS-A) as a measurement tool. The use of GADIS-A scale demonstrates its strong reliability and validity with $\mathrm{Cronbac}h^{\prime }s\;\alpha =0.87$ (56). In this research, the initial “game” project has been modified to “digital media” for examination, with $\mathrm{Cronbac}h^{\prime }s\;\alpha =0.87$ , aligning with Paschke’s research and meeting the reliability criteria outlined in this study (56).

### 3.4 Statistical methods

In this study, the software SPSS 26 was used for the statistical analysis of the collected data. The primary statistical procedures employed encompassed the HARMAN single-factor test to identify potential common-method bias, the Pearson correlation test for examining the correlation between core variables, and linear regression analysis with the least squares method to predict negative emotions using social media addiction, body image comparison, and sleep quality as independent variables. In addition, a chain mediation analysis was conducted to explore the sequential relationship between social media addiction, body image comparison, sleep quality, and negative emotions using SPSS 26 and the PROCESS 3.4 plugins. Finally, the model’s suitability was assessed using Amos28. To determine the total, indirect, and direct effects between the variables, Bootstrapping with a 95% confidence interval was applied, with a significance level of *α* = 0.05.

## 4 Results

### 4.1 Assessment of common-method variance bias

The common-method variance bias pertains to the potential inaccuracies introduced by the measurement technique used. In the context of this research, the deviation in common-method variation may arise from the guidance provided by coaches and the comprehension of measurement issues by young athletes. The HARMAN single-factor test is typically employed to assess the common-method variance deviation. The statistical software SPSS version 28 was used to perform a principal component analysis on variables including digital media addiction, physical appearance comparison, sleep quality, depression, anxiety, and pressure. The Kaiser–Meyer–Olkin (KMO) measure, as shown in Table 2, was confirmed to be KMO = 0.908, surpassing the threshold of 0.6, and the Bartlett test yielded a substantial result with *P* < 0.05. These outcomes indicate the suitability of the questionnaire for factor analysis in this study. Factor analysis revealed nine factors with eigenvalues exceeding 1, with the highest factor contributing 28.060%, which is notably below the critical threshold of 50%. This finding suggests the absence of common-method variance bias in the questionnaire used. Factors such as social media addiction, physical appearance comparison, sleep quality, depression, anxiety, and stress were identified as appropriate for further data exploration and collection.

**Table 2:** Tests by KMO and Bartlett.

KMO value		0.91
Bartlett’s test of Sphericity	Chi-squared approximation	6364.46
	*df*	741
	*p-value*	0.00

### 4.2 Analysis of the correlation between the core variables

The study conducted Pearson correlation analysis to examine the relationships between social media addiction, physical appearance comparisons, sleep quality, and negative emotions. Prior to the analysis, the distribution of the data was assessed using the Kolmogorov–Smirnov (K-S) test due to the sample size exceeding 50. The results, presented in Table 3, indicated that the data exhibited a non-significant *p*-value (>0.05), indicating that the data did not significantly deviate from a normal distribution (or other assumed distribution), thus suitable for further parametric analysis. Subsequent Pearson correlation analysis, detailed in Table 4, revealed significant positive correlations among social media addiction, physical appearance comparisons, sleep quality, and negative emotions, indicating mutual influences among these variables.

**Table 3:** Table of normal distribution test results.

Variable	Sample size	Mean	Standard deviation	Kolmogorov–Smirnov test
Social media addiction	320	38.99	4.61	0.14
Physical appearance comparison	320	11.72	4.83	0.20
Sleep quality	320	3.84	1.62	0.37
Negative emotions	320	30.03	9.22	0.19

* *p* < 0.05, ** *p* < 0.01.

**Table 4:** Results of Pearson’s correlation analysis.

	Social media addiction	Physical appearance comparisons	Sleep quality	Negative emotions
Social media addiction	1			
Physical appearance comparisons	0.18**	1		
Sleep quality	0.26**	0.36**	1	
Negative emotions	0.31**	0.21**	0.49**	1

* *p* < 0.05, ** *p* < 0.01.

### 4.3 Regression analysis of social media addiction, physical appearance comparisons, and sleep quality

The statistics presented in Table 5 show the regression analysis of social media addiction, physical appearance comparison, and sleep quality impacting negative emotions among athletes. Specifically, the regression coefficients for social media addiction (*β* = 0.619), physical appearance comparison (β = 0.396), and sleep quality (β = 0.20755) indicate their individual effects on athletes’ negative emotions. The statistical significance level of *p* < 0.01 confirms the significance of these effect. The information presented in Table 6 indicates that the multiple regression model continues to demonstrate a statistically significant and positive impact, thereby supporting the research hypothesis H1.

**Table 5:** Results of individual regression coefficients.

	Negative emotions						
	*B*	Standard error	*t*	*p*	95% CI	F	R^2^_adj_
Constant	54.21	4.25					
Social media addiction	0.62	0.11	5.73	0.00**	0.41 ~ 0.83	32.88	0.09
Constant	25.39	1.33					
Comparison of Physical Appearance	0.40	0.11	3.78	0.00**	0.19 ~ 0.60	14.28	0.04
Constant	19.44	1.16					
Sleep quality	2.76	0.28	9.91	0.00**	2.21 ~ 3.30	98.15	0.23

* *p* < 0.05, ** *p* < 0.01.

**Table 6:** Results of multiple regression coefficients.

	Regression coefficient	95% CI	Collinearity diagnosis	
			VIF	Tolerance
Constant	35.42** (7.84)	26.57 ~ 44.27	-	-
A digital media addiction	0.89** (3.84)	0.59 ~ 1.19	1.08	0.92
A body comparison scale (4 items)	0.03** (0.36)	−0.16 ~ 0.23	1.16	0.86
A Pittsburgh sleep quality index	2.44** (8.16)	1.85 ~ 3.02	1.20	0.83
Sample size	320			
R2	0.27			
Adjusted R2	0.26			
*F*-value	*F* (3,316) = 39.17, *p* = 0.00			

### 4.4 Chain mediation between social media addiction, body appearance comparison, sleep quality, and negative emotions

Taking social media addiction as an independent variable and negative emotions of young athletes as a dependent variable, and body appearance comparison and sleep quality as mediating variables, a chain mediation model was employed to examine how social media addiction impacts negative emotions through physical appearance comparison and sleep quality. Data analysis was conducted using SPSS 23 and PROCESS 3.4 plugins, while demographic variables such as gender (with males coded as 1 and females as 2), age, and athlete level were controlled for. Gender, age, and athlete level were included in the analysis as covariates to restrict the influence of these variables in the examination of social media addiction’s impacts on adverse emotional states. With negative emotions of adolescent athletes as the dependent variable, regression analysis was used to evaluate the impact of social media addiction on negative emotions in adolescent athletes. The results are presented in Table 7. The results indicate a substantial positive correlation between social media addiction and negative emotions among athletes (*β* = 0.202, *p* < 0.01), suggesting that higher levels of social media addiction were associated with increased negative emotions in adolescent athletes. This finding supports the study’s main conclusion that social media addiction contributes to heightened negative emotions in adolescent athletes (see Table 8).

**Table 7:** Results of the mediating effect model test.

	Comparison of physical appearance		Sleep quality		Negative emotions		Negative emotions	
	*B*	*t*	*B*	*t*	*B*	*t*	*B*	*t*
Constant	17.876**	6.75	3.35**	4.17	51.54**	10.76	38.42**	8.00
Gender	0.81	1.46	1.12**	7.12	4.09**	4.07	1.08	1.08
Age	0.07	0.72	0.07**	2.70	0.02	−0.11	−1.38	1.38
Sports level	−0.31	−0.61	0.07	0.46	0.55	−0.60	−0.76	0.76
Social media addiction	0.20**	3.41	0.09**	5.19	0.67**	6.22	0.39**	3.77
Comparisons of physical appearance			0.10**	6.14			0.02**	0.23
Sleep quality							2.50**	7.60
*R* ^2^	0.04		0.32		0.14		0.29	
Adjusted *R*^2^	0.03		0.31		0.13		0.28	
*F*-value	*F* (4,315) = 3.443,*p* = 0.00		*F* (5,314) = 29.825,*p* = 0.00		*F* (4,315) = 12.802,*p* = 0.00		*F* (6,313) = 21.276,*p* = 0.00	

* *p* < 0.05, ** *p* < 0.01.

**Table 8:** Results analysis of mediating benefits.

Term	Effect	Boot SE	BootLLCI	BootULCI	*z*	*p*
Social media addiction ⇒ Physical appearance comparison ⇒ Negative emotions	0.01	0.01	0.00	0.01	0.47	0.01
Social media addiction ⇒ Sleep quality ⇒ Negative emotions	0.22	0.03	0.06	0.17	8.39	0.00
Social media addiction ⇒ Physical appearance comparisons ⇒ Sleep quality ⇒ Negative emotions	0.04	0.01	0.01	0.05	5.32	0.00

BootLLCI refers to the lower limit of the 95% interval for Bootstrap sampling, and BootULCI refers to the upper limit of the 95% interval for Bootstrap sampling.

**Figure 2:**
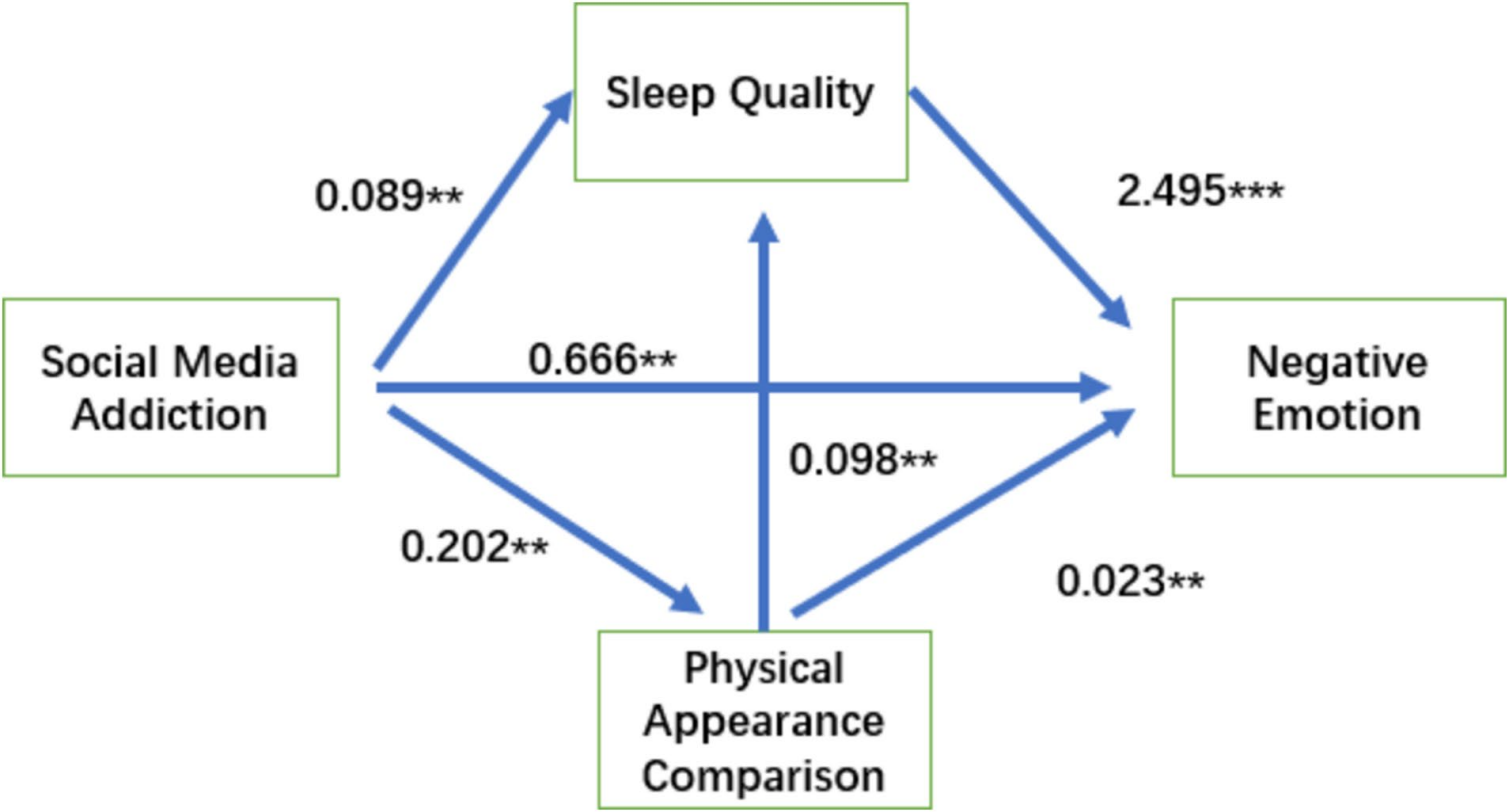
Graph of chain-mediated effects of body appearance and sleep quality.

To further examine the mediating influence of body appearance and sleep quality, these variables were incorporated into the analysis. Two parallel mediating pathways were presumed: the first being “social media addiction ⇒ body appearance comparison ⇒ negative emotion” and the second being “social media addiction ⇒ sleep quality ⇒ negative emotion. In addition, a mediated chain pathway was established, denoted as “social media addiction ⇒ body appearance comparison ⇒ sleep quality ⇒ negative emotion.” The results revealed that the effect size in the parallel mediation of “social media addiction ⇒ body appearance comparison ⇒ negative emotion” was Effect = 0.05 (*p* = 0.006 < 0.01). The 95% BootCI interval of the indirect effect did not contain 0, signifying the presence of this mediating effect and confirming hypothesis H2. Similarly, the effect size of the parallel mediating pathway “social media addiction ⇒ sleep quality ⇒ negative emotion” was Effect = 0.05 (*p* = 0.000 < 0.01). BootCI value of the 95% confidence interval for the indirect effect excluded 0, indicating the existence of this mediating effect and validating hypothesis H3. The effect size of the chain mediating pathway “social media addiction ⇒ body appearance comparison ⇒ sleep quality ⇒ negative emotion” was Effect = 0.039 (*p* = 0.000 < 0.01). The 95% BootCI interval of the indirect effect did not contain 0, demonstrating the presence of this mediating effect and thereby confirming the validity of hypothesis H4. Consequently, it is evident that social media addiction not only impacts the negative emotions of adolescent athletes directly, positively, and considerably but also reinforces its influence through body appearance comparison, the parallel mediation of sleep quality, and chain mediation. By considering the regression path coefficient of the chain mediation, a pathway model illustrating the influence of negative emotions stemming from social media addiction on adolescent athletes was established, as illustrated in Figure 2.

## 5 Discussion

### 5.1 The influence of social media addiction on adverse emotional experiences among adolescent athletes

Research has indicated that addiction to social media has a notable and positive influence on the negative emotions experienced by adolescent athletes. The deeper the dependence on social media, the more pronounced the impact on the adverse emotions experienced by adolescent athletes. Summarized meta-analysis reviews have demonstrated a substantial effect of social media on the happiness and negative emotions of adolescents (57). The emotional well-being of adolescent athletes is adversely affected by social media addiction, leading to feelings of anxiety, depression, and stress. These young athletes encounter substantial pressure from various sources such as coaches, parents, peers, and their expectations to excel in competitions and training (58). In particular, constant exposure to and comparison with the accomplishments and successes of other athletes on social media exacerbate this pressure. Consequently, adolescent athletes may develop doubts about their abilities and feel compelled to continuously achieve higher standards, resulting in heightened anxiety and depression (59). Concerns about meeting external expectations or losing control over competitions contribute to increased psychological stress. Although social media offers a platform for athletes to connect and share experiences, excessive usage can hinder real-life social interactions and team cohesion (60). In team sports, face-to-face communication and collaborative activities are crucial for fostering trust, camaraderie, and team spirit. Substitution of social media engagement diminishes actual team interactions among athletes, thereby reducing emotional bonds and support networks within the team. This lack of connection can lead to feelings of loneliness and isolation, intensifying anxiety and depression (61). Adolescent athletes are susceptible to negative feedback, criticism, and cyberbullying on social media (61), which are prevalent hazards contemporary athletes encounter. For adolescents who are in the process of cultivating and defining their self-awareness, receiving negative feedback can impact their self-esteem and self-worth considerably. This can result in lasting psychological distress, thereby increasing the likelihood of anxiety and depression, and affecting both mental well-being and athletic performance. Social media addiction poses challenges for young athletes in managing their time effectively, affecting their training, academic responsibilities, and rest periods. Excessive social media use diminishes the time available for training, schoolwork, and other activities essential for physical and mental growth. This imbalance in priorities can lead to a decline in athletic performance and engender feelings of guilt and anxiety about time management failures. Moreover, difficulties in managing time effectively may elevate stress levels and make athletes feel overwhelmed, thereby affecting their mental health.

### 5.2 Mediating effect of physical appearance comparison on adverse emotional consequences experienced by adolescent athletes because of their addiction to social media

This study illustrates that the comparison of physical appearance has a crucial and comprehensive mediating function in the influence of social media dependency on the adverse emotions experienced by teenage athletes. Social media addiction intensifies the negative emotions of adolescent athletes through the act of comparing their physical appearance. Social media platforms are saturated with idealized body images and athletic achievements, leading to frequent comparisons between adolescent athletes and these idealized standards. Research has shown that frequent body comparisons on social media are linked to substantial decreases in body satisfaction (62). For athletes, striving for ideal standards can result in reduced satisfaction with their bodies and negative self-image. Consequently, this impacts their motivation to train and their performance in competitions, subsequently heightening negative emotions such as anxiety and depression. Unrealistic body ideals and expectations, often portrayed through carefully curated and edited images on social media, can prompt teenage athletes to set unattainable body image standards. Holland et al. found that social media use is associated with unrealistic expectations regarding athletic performance and physical appearance (63). Pursuing these unattainable standards can lead to persistent frustration and self-doubt, contributing to increased feelings of depression and anxiety. The conflict between an athlete’s identity and their sense of self-worth is also a substantial concern. For adolescent athletes, their identity as an athlete is a crucial component of their self-concept. Comparisons of physical appearance on social media can raise doubts about self-worth and identity. Research by Vandenbosch has shown that comparing their appearance with others on social media can impact an individual’s self-concept, particularly concerning core aspects of their identity such as their athletic identity (64). Vacillations in self-concept can lead to heightened internal conflict, resulting in emotional instability and an increase in negative emotions. Furthermore, there is an imbalance between social feedback and social support. Social media platforms offer young athletes a space to showcase their accomplishments and receive feedback. However, focusing excessively on comparisons of physical appearance can lead athletes to overly prioritize social feedback, particularly when it is negative or related to body image. This can affect the quality and quantity of social support they receive on social media. As found in studies by Stapleton, this indicates that negative feelings stemming from comparisons on social media are linked to lower levels of social support and increased symptoms of depression (65). The lack of adequate social support can degrade athletes’ ability to cope with the stress of training and competition, leading to heightened negative emotions. The mediating role of physical appearance comparison in the negative emotional consequences of social media addiction on adolescent athletes increases their likelihood of experiencing negative emotions such as anxiety and depression. This is primarily due to diminished body satisfaction, unrealistic physical goals and expectations, conflicts related to athlete identity and self-worth, and an imbalance in social feedback and support.

### 5.3 Mediating effect of sleep quality on negative emotional effects of social media addiction among adolescent athletes

This study illustrates that sleep quality serves as a substantial and comprehensive mediator in the relationship between social media addiction and adverse emotions experienced by adolescent athletes. Social media addiction intensifies negative emotions among adolescent athletes by influencing their sleep quality. The quality of sleep is closely associated with the capacity for recovery. Sleep is a crucial element in an athlete’s recovery process, which is vital for their training and competitive performance. Social media addiction diminishes both the duration and quality of sleep (66), directly affecting the physical and mental recovery of athletes directly (67). The quality of sleep of athletes is inextricably linked to their emotional well-being, cognitive abilities, and physical performance on the following day. Sleep disturbances triggered by social media indirectly heighten negative emotions such as anxiety and depression in athletes by impeding their recovery capabilities. Sleep also plays a role in emotional regulation. Inadequate sleep quality, which is characterized by difficulties in falling asleep, interruptions during sleep, or insufficient sleep, has been demonstrated to hinder an individual’s capacity to regulate emotions (41). Furthermore, poor sleep quality is associated with challenges in emotional regulation, resulting in more frequent and intense negative emotional episodes. For adolescent athletes with social media addiction, nighttime social media use disrupts sleep, affects emotional stability, and hampers their ability to manage training stress the following day (68). Sleep deprivation affects cognitive functions in athletes, including attention, decision-making, and memory, which are crucial for athletic performance, particularly in sports requiring precise skills and strategic decision-making (69). Sleep deprivation can influence cognitive function and emotional well-being considerably. Sleep-related issues stemming from social media addiction impact not only athletes’ physical well-being but also their mental health, diminishing the cognitive processing required for training and competition while acting to amplify negative emotions. A detrimental cycle exists between social media use and sleep quality. Nighttime social media engagement disrupts sleep due to light exposure and psychological arousal. Subsequently, a lack of sleep heightens stress levels the next day, prompting increased social media use as a coping mechanism. There is a positive correlation between sleep disturbances and daytime stress levels (70). The adverse cycle of social media use and its effects on sleep quality contribute to the negative emotions experienced by adolescent athletes. Given the stress associated with training and competition, sleep issues arising from social media addiction may further compound this stress and exacerbate negative emotions. The mediating role of sleep quality in the impact of social media addiction on the negative emotions of adolescent athletes is evident in reduced recovery, difficulties with emotional regulation, impaired cognitive function, and negative cycles involving social media use, sleep patterns, and stress levels.

### 5.4 The mediating effects of body appearance comparison and sleep quality on the chain of social media addiction and negative emotion among adolescent athletes

This study illustrates the substantial chain mediating effects of physical appearance comparison and sleep quality on social media addiction and negative emotions among adolescent athletes. The interconnected relationship between physical appearance comparisons, compromised sleep quality, social media addiction, and negative emotions in young athletes constitutes a multifaceted psychological and physiological mechanism that influences the emotional well-being of this cohort. The pervasive promotion of ideal body images on social media platforms serves as a catalyst for young athletes to engage in frequent comparisons of their body appearance. Research by Fardouly has indicated that such comparisons are often linked to increased body image dissatisfaction (60, 62). This dissatisfaction and the resulting negative emotions can, in turn, impact the sleep quality of adolescent athletes. Studies have shown that psychological stress and emotional distress are influential factors in sleep disturbance (71). Anxiety and body image dissatisfaction can intensify difficulties in falling asleep, interruptions during sleep, or insufficient sleep. Sleep disturbances intensify negative emotions throughout the day, creating a vicious circle. Adequate sleep is crucial for the physical and mental recovery of adolescent athletes because poor quality sleep can directly impair emotional regulation and adaptation to the demands of training and competition. Sleep deprivation can affect the brain’s emotional processing areas, heighten sensitivity to negative emotions, and diminish the experience of positive emotions (72). Consequently, sleep problems induced by social media use may directly contribute to increased anxiety, depression, and other negative emotions. Social media addiction can initially elevate psychological stress and dissatisfaction by promoting frequent comparisons of physical appearance, ultimately disrupting sleep quality and indirectly increasing the likelihood of negative emotions. This chain-mediated effect underscores the intricate network of interactions between social media addiction, body image dissatisfaction, sleep problems, and negative emotions that collectively influence the mental well-being of adolescent athletes.

## 6 Conclusions and prospects

### 6.1 Conclusion

Initially, excessive engagement with social media platforms has a notable influence on the adverse emotional states experienced by young athletes, indicating that heightened social media use correlates directly with elevated negative emotions such as anxiety and depression among this demographic.

Furthermore, the comparison of people’s physical appearance serves as a substantial intermediary factor between the use of digital media and the psychological well-being of adolescent athletes. This implies that social media platforms facilitate the comparison of idealized body images, worsening discontent and negative emotions regarding one’s physical attributes.

Moreover, sleep quality acts as a crucial intermediary factor between digital media consumption and the mental health of adolescent athletes. Sleep quality functions as a pivotal mediation pathway through which the use of digital media affects the psychological well-being of young athletes. The use of social media platforms influences sleep patterns and intensifies negative emotions by disrupting sleep quality.

Finally, the comparison of physical appearance and sleep quality collectively serves as a chain of intermediary factors between social media dependency and negative emotions. This indicates that the comparison of physical attributes and sleep quality are interconnected and jointly contribute to the emotional health of adolescent athletes.

### 6.2 Research prospects

This study addresses the mediating effects chain between social media addiction, body appearance comparison, sleep quality, and negative emotions in young athletes. It offers a new perspective for understanding how these factors interact. Despite the limitations of this study, several future research directions are suggested. First, a longitudinal study with extended follow-up periods is recommended to identify the causal links between social media use, body image comparison, sleep quality, and negative mood. Second, a multicultural investigation is proposed to explore how cultural factors impact the mental well-being of adolescent athletes. Third, the study advocates the creation and assessment of intervention tactics, namely, designing and implementing interventions aimed at reducing social media addiction, improving body image cognition and improving sleep quality, and evaluating the effect of these measures on improving the mental health of young athletes. In addition, a comprehensive examination is suggested to further explore the psychological and physiological mechanisms underlying the influence of social media use, body image concerns, and sleep disturbances on the emotions and behaviors of adolescent athletes and to analyze their interplay. It is anticipated that this study will deepen our understanding of social media addiction and its implications for the mental health of adolescent athletes, providing a scientific foundation for the formulation of preventive and intervention measures. By conducting thorough investigations in these areas, the goal is to establish a more robust theoretical framework and offer practical guidance to improve the mental well-being of adolescent athletes.

## Data Availability

The original contributions presented in the study are included in the article/supplementary material, further inquiries can be directed to the corresponding author.
